# Michigan Dental Association

**Published:** 1897-12

**Authors:** Henry Raymond


### PROCEEDINGS.

### Michigan Dental Association.

TUESDAY, JUNE 8, 1897.

Meeting was called to order by President Diack at 2:30 P.M., and was opened with prayer by the Rev. Louis Brown. The address of welcome was given by the Mayor, A. T. Metcalf, and responded to by Dr. J. Ward House, who moved that a rising vote of thanks be tendered the Mayor for his very hearty welcome to Battle Creek. The motion was seconded and responded to unanimously. Vice-President Harvey then took the chair while President Diack read his address. Dr. Metcalf, Chairman of the Executive Committee gave his report. Dr. Harvey's motion that it be accepted was carried. Dr. Blackmarr as Chairman of Publication Committee then rendered his report. Dr. Harvey moved that the report of the Publication Committee be laid on the table, and a committee be appointed to confer with Dr. Hoff as Dr. Taft's representative, and report at 7:30 in the evening. Dr. Fowler moved that this committee be empowered to act and employ a reporter. Motion supported and carried.

The President appointed on the committee, Drs. Blackmarr, House and S. M. White. Dr. Hoff then moved that a committee be appointed for revision and action on the President's address. Motion seconded and carried.

Dr. Harvey moved that a committee on resolutions be appointed. Motion seconded and carried. Meeting then adjourned.

TUESDAY EVENING, JUNE 8, 1897.

Meeting was called to order at 7:50 P. M., by President Diack. Minutes of the Afternoon meeting were read and approved. Dr. House, Chairman of the Committee appointed to confer with Dr. Hoff in regard to reporting the meeting, reporded that they had entered into an arrangement to employ a reporter, and Dr. Hoff on behalf of Dr. Taft would be responsible for the expense. Dr. Rex then moved that the offer of Dr. Hoff to furnish a reporter be accepted. Motion supported and carried. Dr. A. F. Monroe, of Flint, then read a paper on "Treatment of Abscess." The discussion was opened by Dr. Porter, of Petoskey, and continued by Drs. Williams, House, Monroe, Young, Morgan, Wood, Fowler and Thompson. Subject was closed by Dr. Monroe. Dr. R. W. Morse, of Lansing, read a paper entitled "The Point of Contact," discussion opened by Dr. J. L. Sweetnam, of Manistee, and continued by Drs. Hoff, J. L. Young, Lowrey, of Kansas City, Field, Loeffler, House, Rix and Parker. Dr. Morse then closed the subject and it was passed. The President then appointed the following committees: on Resolutions, Drs. Parker, Morgan and Rix: on reporting and revision of President's address, Drs. Field, Hoff and Loeffler. The roll was called at this session.

WEDNESDAY MORNING, JUNE 9, 1897.

The President called the meeting to order at 9:45 P. M., Dr. H. T. Harvey, of Battle Creek, read a paper entitled, "To what extent and when are we justified in using Cataphoresis? Is there danger of injuring the Dental pulp or other Tissues by its uses?" The paper was discussed by Drs. Morse, of Lansing, and Blackmarr, of Jackson, and general discussion was taken part in by Drs. Siddal, Fritz, Loeffler, H. C. Raymond and Field, and Dr. Harvey closed the discussion.

Dr. E. T. Loeffler, of Saginaw, read a paper on "Dental Bacteriology," which was discussed by Drs. Codding, of Dowagiac, and Dr. Hoff, of Ann Arbor. Meeting then adjourned.

WEDNESDAY AFTERNOON, JUNE 9, 1897.

Dr. Diack called the session to order at 3:00 P. M. The

Board of Censors reported favorably on the application of twenty-two dentists for membership in the Association. Dr. Hoff moved that the Secretary cast a unanimous ballot for their election. Dr. J. H. Kellogg then read a paper entitled, "The Chemistry and Bacteriology of the Stomach in relation to Dental Caries."

Dr. N. S. Hoff opened the discussion and was followed by others. Dr. Hoff moved that a hearty vote of thanks be given Dr. Kellogg for his very able paper. Unanimously carried.

WEDNESDAY EVENING, JUNE 9, 1897.

Meeting was called to order by President Diack at 9:00 P. M. Before the general order of business was commenced, Dr. E. C. Moore exhibited a new lathe and method of making matrix for shell crowns. Dr. Lowrey, of Kansas City, exhibited a new method of Crown and Bridge work. Secretary read the minutes of the annual meeting of the Board of Directors which were approved. The Treasurer read his annual report which was referred to the Auditing Committe. Dr. W. L. Williams, of Sault. Ste. Marie, offered a resolution recommending Dr. George H. Mosher, of Jackson, for reappointment on the Board of Dental Examiners. Motion to adopt was carried and a motion that it be signed by the officers of the Association and forwarded to the Governor was also sustained.

Dr. J. L. Sweetnam moved that a Committe of five be appointed by the chair to confer with other Committees of the Tri-State meeting regarding time and place of the meeting in 1898. Motion supported and carried. Dr. J. H. Brown, of Port Huron, read an invitation from the Mayor of Port Huron, and the dentists there to hold the meeting of 1898, in that city. A motion was carried to hold the annual meeting in Port Huron in 1899. As the meeting in 1898 will be held as a Tri-State with Ohio and Indiana.

Dr. Parker, of Grand Rapids, Chairman of the Committee on resolutions offered resolutions on the deaths of Drs. Jerry A. Robinson, of Jackson, H. W. Ray, St. Joseph, William Gooding, of Saginaw, as follows: Your Committee on Resolutions beg to report that in the death of our Brothers Drs. Jerry A. Robinson, William Gooding and H. W. Ray, we recognize the loss that this association has sustained in the silence never to be broken of those where words of wisdom and council have for many years been an inspiration and guide to so many of us; and that—while recognizing that in the unvarying laws of nature it is just as natural to die as to be born. Still the passing from among us, with whom we have been so long associated, always comes to us with a mental shock, no matter what the time or manner of their death. Still, when a life has been as fully rounded out as that of our dear old uncle Jerry—as we loved to call him—when old age had set its limits upon his further activities, his going out is but the fulfillment of nature's plan; and we accept, with bowed heads, this inevitble end. Still, let us remember that "he lives long, who lives well," but he who lives both long and well, has given to the world a doubled portion of human effort.

In the death of Brother Ray passing out from among us suddenly in the prime of life, his life's work half unfinished; it seems as though "nature was out of joint," and we rebel against her unfulfilled promise. But in all this, let us try to remember that no life is short that has been devoted to the good of others. And then we, as members of this association, do hereby desire to express our gratitude for all that our brothers have given to us of their wisdom, council and brotherly love, and that these resolutions be made a part of the records of this association.

J. C. Park,
T. G. Rix,
W. R. Morgan.

Motion was passed adopting the resolution by a unanimous rising vote. Dr. G. L. Field, Chairman of Committee on President's address, presented his report which was accepted. Motion was carried to adopt the report.

REPORT OF COMMITTEE APPOINTED TO REVISE AND REPORT ON THE RECOMMENDATIONS OF THE PRESIDENT'S ADDRESS.

*Resolved:* That it is the desire of the Michigan State Dental Association, that local or district dental societies should be organized in favorable localities, which shall hold meetings at convenient times and places, and shall be somewhat informed as government and proceedings, aiming rather to cultivate social and professional relations, and to have a watch-care of professional interest in the several localities where organized, than to the consideration of scientific or artistic subjects of professional interest. The hope being that all such organizations shall strive to increase the attendance and interest of the annual State meeting.

*Resolved:* That whereas it has come to the notice of the association that some of its members have engaged the public prints as well as other unfair methods to call attention to their superior advantages and abilities as an inducement to secure patronage; we therefore, again state with emphasis, that all such practices are not only unprofessional but in direct violation of our code of ethics. Indulgence in such practices will be sufficient cause for expulsion of a member of this association, and no one indulging in such practices shall be eligible to membership in this association.

Signed,

GEO. L. FIELD,
E. T. LOEFFLER,
N. S. HOFF.

The election of officers was next in order and resulted as follows: President, E. T. Loeffler, Saginaw; First Vice President, H. T. Harvey, Battle Creek; Second Vice President, Henry C. Raymond, Detroit; Secretary, S. M. Fowler, Muskegon; Treasurer, George H. Mosher, Jackson; Member of Board of Censors, F. H. Codding, Dowagiac.

Adjourned.

THURSDAY, JUNE 10, 1897.

President Diack called the meeting to order at 9:45 A. M., Dr. N. S. Hoff read a paper entitled, "How to establish a Dental Practice." Dr. C. B. Blackmarr, Jackson, read a paper entitled, "How to conduct a City Practice." Dr. Burt Abell followed with a paper entitled, "How to conduct a Country Practice." These papers were discussed by Doctors A. T. Metcalf, James Cleland, Essig, George L. Field, J. Taft, N. S. Hoff, S. M. Fowler, H. S. Lowrey, Kansas City; Dr. Fritz, C. P. Wood and W. A. Douglas. Dr. Hoff then made a motion that a committee be appointed to confer with Dr. J. H. Kellogg and cooperate with him in the work embodied in the paper he read before the Association. Motion was supported and carried, and the President appointed on that committee, Doctors Castle, Battle Creek; E. T. Loeffler, Saginaw; A. W. Haidle, Ann Arbor; and H. T. Harvey, of Battle Creek. Motion was passed that the matter referring to the publication of the proceedings in a separate volume or in two issues of the DENTAL REGISTER, be left in the hands of the Publication Committee with power to act. Dr. Hoff moved that a further discussion of the papers read in the morning session be carried on in the afternoon. Dr. Hoff then made a motion that we adjourn to meet at the call of the President.

THURSDAY AFTERNOON, JUNE 10, 1897.

Meeting was called to order by the President at 4 P. M. Dr. Harvey moved that the subject of the papers read at the morning session be passed. Supported and carried. Minutes of previous meetings were read and approved. The Board of Censors reported favorably on three applications for membership, and Dr. Hoff moved that the Secretary cast a unanimous ballot for their election. Motion was supported and carried. The President appointed the following on the Committee to confer with the other State Committee of the Tri-State meeting, as to time and place of meeting in 1898: E. T. Loeffler, Saginaw; S. M. Fowler, Muskegon; H. C. Raymond, Detroit; S. M. White, Benton Harbor; J. L. Sweetnam, Manistee. Dr. A. T. Metcalf then gave a talk on the State Dental Law and a general discussion followed. Dr. Blackmarr the only member of the Legislative Committee present, reported, and a motion was carried to accept the report and discharge the committee. A discussion then took place regarding the advisability of having one or several on a committee to see that the dental law be enforced. Dr. Abell moved that a committee of one be appointed to confer with and see that the Prosecuting Attorneys in their several districts prosecute all illegal practitioners. Motion was seconded. There were other motions made which were not supported. Dr. House moved an amendment that all complaints against illegal practitioners be made to the Secretary of the Association, he to take charge of such and jointly with the President act accordingly and see that such cases be placed in the proper hands for prosecution. This motion was supported and carried. Dr. A. T. Metcalf promised to aid in whatever way he could, those who would have this work in hand.

The Board of Directors then offered the following resolutions, that Dr. B. H. Lee, of Grand Rapids, be suspended from membership in this Association for one year for violation of the code of ethics, and that Dr. H. P. Snyder, of Grand Rapids, be expelled from this Association for violation of the code of ethics. After considerable discussion the resolution was adopted.

The following were elected to membership: Clarence W. Young, Allegan; Edward B. Spalding, Detroit; S. Straith, Lakeview; F. E. Morey, Hillsdale; W. A. Douglas, Bronson, Branch Co.; C. E. Speer, Jonesville; Wilsie D. Reed, Tecumseh; Arthur C. Runyan, South Haven; Edward D. Slawson, Bay City; H. E. Hooper, Decatur; A. L. Le Gee, Athens; G. W. Lyons, Jackson; W. D. Geiger, Alpena; P. P. Nelson, Wyandotte; Geo. A. Wilkinson, Bangor; E. H. Coller, Battle Creek; W. M. Welet, Homer; R. P. James, Kalamazoo; W. S. Hinkley, Hartford; E. P. Backus, St. Joseph; Frank H. Honey, Charlotte; M. Westbrook, Kalamazoo; A. E. Cottingham, Ionia; S. J. Farnum, Cassopolis; Abram Polham, Plymouth and B. Griffin Miller, Ionia.

The Auditing Committee then reported that they had found the Treasurer's report and the several bills presented, correct. Dr. Hoff moved that the Treasurer be authorized to pay the several amounts. Motion supported and carried. Meeting then adjourned.

HENRY RAYMOND, *Secretary*.

